# Increasing Signal Specificity of the TOL Network of *Pseudomonas putida* mt-2 by Rewiring the Connectivity of the Master Regulator XylR

**DOI:** 10.1371/journal.pgen.1002963

**Published:** 2012-10-11

**Authors:** Aitor de las Heras, Sofia Fraile, Victor de Lorenzo

**Affiliations:** Systems Biology Program, Centro Nacional de Biotecnología, Consejo Superior de Investigaciones Cientificas, Madrid, Spain; Universidad de Sevilla, Spain

## Abstract

Prokaryotic transcription factors (TFs) that bind small xenobiotic molecules (e.g., TFs that drive genes that respond to environmental pollutants) often display a promiscuous effector profile for analogs of the *bona fide* chemical signals. XylR, the master TF for expression of the *m-*xylene biodegradation operons encoded in the TOL plasmid pWW0 of *Pseudomonas putida*, responds not only to the aromatic compound but also, albeit to a lesser extent, to many other aromatic compounds, such as 3-methylbenzylalcohol (3MBA). We have examined whether such a relaxed regulatory scenario can be reshaped into a high-capacity/high-specificity regime by changing the connectivity of this effector-sensing TF within the rest of the circuit rather than modifying XylR structure itself. To this end, the natural negative feedback loop that operates on *xylR* transcription was modified with a translational attenuator that brings down the response to 3MBA while maintaining the transcriptional output induced by *m-*xylene (as measured with *a luxCDABE* reporter system). XylR expression was then subject to a positive feedback loop in which the TF was transcribed from its own target promoters, each known to hold different input/output transfer functions. In the first case (*xylR* under the strong promoter of the *upper* TOL operon, *Pu*), the reporter system displayed an increased transcriptional capacity in the resulting network for both the optimal and the suboptimal XylR effectors. In contrast, when *xylR* was expressed under the weaker *Ps* promoter, the resulting circuit unmistakably discriminated *m-*xylene from 3MBA. The non-natural connectivity engineered in the network resulted both in a higher promoter activity and also in a much-increased signal-to-background ratio. These results indicate that the working regimes of given genetic circuits can be dramatically altered through simple changes in the way upstream transcription factors are self-regulated by positive or negative feedback loops.

## Author Summary

It is generally taken for granted that promoters regulated by transcriptional factors (TFs) that respond to small molecules control their specificity to given effectors by tightening or relaxing the intrinsic dual interaction between the TF and the particular inducer. One such promoter is *Pu*, which drives expression of an operon for the biodegradation of *m-*xylene by the soil bacterium *P. putida* mt-2. While XylR, the chief TF of this system, binds this substrate and activates *Pu*, the same regulator responds, to a lesser extent, to 3-methylbenzylalcohol and thus also activates the promoter. This work provides evidence that such natural effector promiscuity of the system can be altogether suppressed by replacing the naturally occurring negative autoregulation loop that governs XylR expression with an equivalent positive feedback loop. Based on this result, we argue that signal specificity of a given regulatory device depends not only on the TF involved but also on TF connectivity to upstream signals and downstream targets.

## Introduction

**Figure 1:**
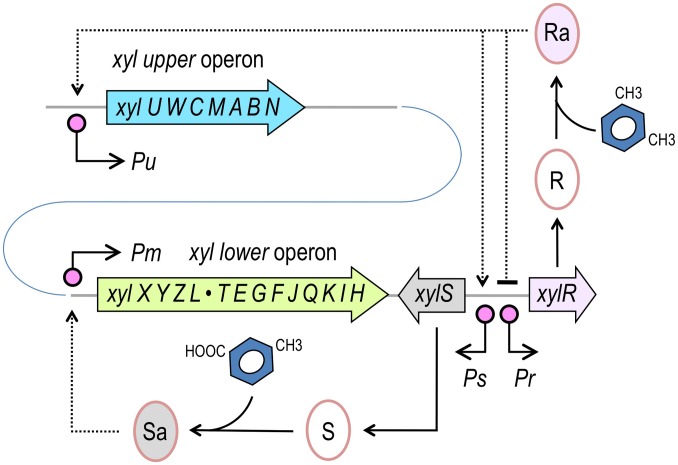
Structure of the TOL network of *P. putida* mt-2. The TOL pathway encompasses two different operons, the *upper* operon, (*xylUWCMABN*), the products of which transform *m*-xylene into 3-methylbenzoate, and the *lower* operon (*xylXYZLTEGFJQKIH*) that produces enzymes for further metabolism of this compound into TCA cycle intermediates. XylR and XylS are the transcriptional regulators that control the expression of either operon. The master regulatory gene *xylR* is encoded in a location adjacent to the end of the *lower* operon and is expressed from the *Pr* promoter. XylR is produced in an inactive form (R) that, in the presence of the pathway substrate (*m*-xylene) or pathway intermediates, such as 3-methylbenzyl alcohol (3MBA), changes to an active form (Ra). XylRa then activates both *Pu* and *Ps*, triggering expression of the *upper* pathway and XylS, respectively. At the same time, XylRa acts as repressor of its own transcription, thereby decreasing its own expression. In the absence of *m*-xylene, XylS is produced at low levels and changes from the inactive form (S) to an active state (Sa) by binding 3-methylbenzoate. In turn, XylSa is able to induce expression of the *meta* pathway by activating the *Pm* promoter (note that operons and regulatory elements not to scale).

The mechanisms that bacteria use to transduce external stimuli into specific responses rely on connected transcriptional factors that shape circuit-like input/output devices [1]. Such networks are comprised of interacting molecular components and can adopt different topologies [2]. The responses of a specific regulatory network to given stimuli are then fine-tuned by the dynamics of its interacting constituents [3], [4]. Free-living bacteria have evolved to respond and adapt to the perturbations derived from a fluctuating environment by increasing the complexity of their regulatory circuits [5]. The TOL plasmid pWW0 of the soil bacterium *Pseudomonas putida* mt-2 is a good example. This plasmid encodes two catabolic operons for biodegradation of *m-*xylene [6] that are subject to an intricate regulatory control involving the interplay among various transcription factors (TFs) [7], [8]. The master regulatory element of the system is the σ^54^-dependent regulator XylR, which, in the presence of its natural inducers, acts on the *Pu* promoter of the *upper* TOL operon. In addition, XylR triggers the expression of the gene that encodes a second regulator, XylS, via the *Ps* promoter [9]. Due to the divergent character of the *Ps* and the *Pr* promoters (driving expression of *xylR*, Figure 1), the activation of the *Ps* promoter not only triggers the expression of *xylS* but also leads to the down-regulation of *xylR* transcription [10]. XylR is optimally activated by the primary substrates of the TOL system, such as *m*-xylene or toluene. However, this TF is not entirely specific for these effectors, as it also responds to a large number of structural analogs. These analogs include both non-substrates as well as metabolic intermediates of *m*-xylene biodegradation, e.g., 3-methylbenzylalcohol (3MBA) [6], [11], resulting in a degree of naturally occurring effector promiscuity. The transcriptional output produced by XylR on the target promoters *Pu* and *Ps* is in turn limited by intracellular concentrations of the TF [12], [13] and σ^54^ [14]. This extant configuration of the system not only leads to a quick response to XylR effectors when cells enter the stationary phase, but it also restricts the *Pu* promoter to low capacity i.e. poor maximum output. The existing characteristics of the XylR-based regulatory network have likely evolved for adjusting the tradeoff between transcriptional efficiency and physiological burden in the natural context, constraining the output of the system. This natural control of the *xylR* expression loop in the context of the TOL plasmid limits the value of the system as the primary component of whole-cell biosensors [15], [16]. Previous attempts to increase the performance of XylR/*Pu*-based biosensing devices have included *in vitro* evolution of the TF [17], [18], construction of regulatory cascades [19] and improvement of the ribosome binding sequence (RBS) of the reporter genes [20]. None of these approaches, however, solve the problem of effector promiscuity. The issue at stake is, therefore, whether we can artificially change such an effector-relaxed/low-output circuit regime into a high-signal specificity/high-capacity counterpart without modifying the XylR protein.

In this work, we report one strategy to overcome the constraints imposed by the natural architecture of the TOL network on the function of the XylR/*Pu* regulatory node of the plasmid. To this end, we adopted a *Pu-luxCDABE* reporter integrated into the chromosome of *P. putida* for accurately measuring system performance. In this genetic background, we then designed and tested various combinations of translation signals, promoter strengths and regulatory loops aimed at [i] suppressing the effect of effector promiscuity on XylR/*Pu* output and [ii] enhancing the response to optimal inducers (e.g., *m*-xylene). The results herein demonstrate that the working regimes of regulatory nodes, including their signal specificity, can be dramatically altered by changing the upstream connectivity of the TFs involved in the network instead of mutating the structure of the corresponding proteins.

## Results

### Monitoring activity of the *Pr/xylR/Pu* regulatory loop with a formatted *P. putida* reporter strain

**Figure 2:**
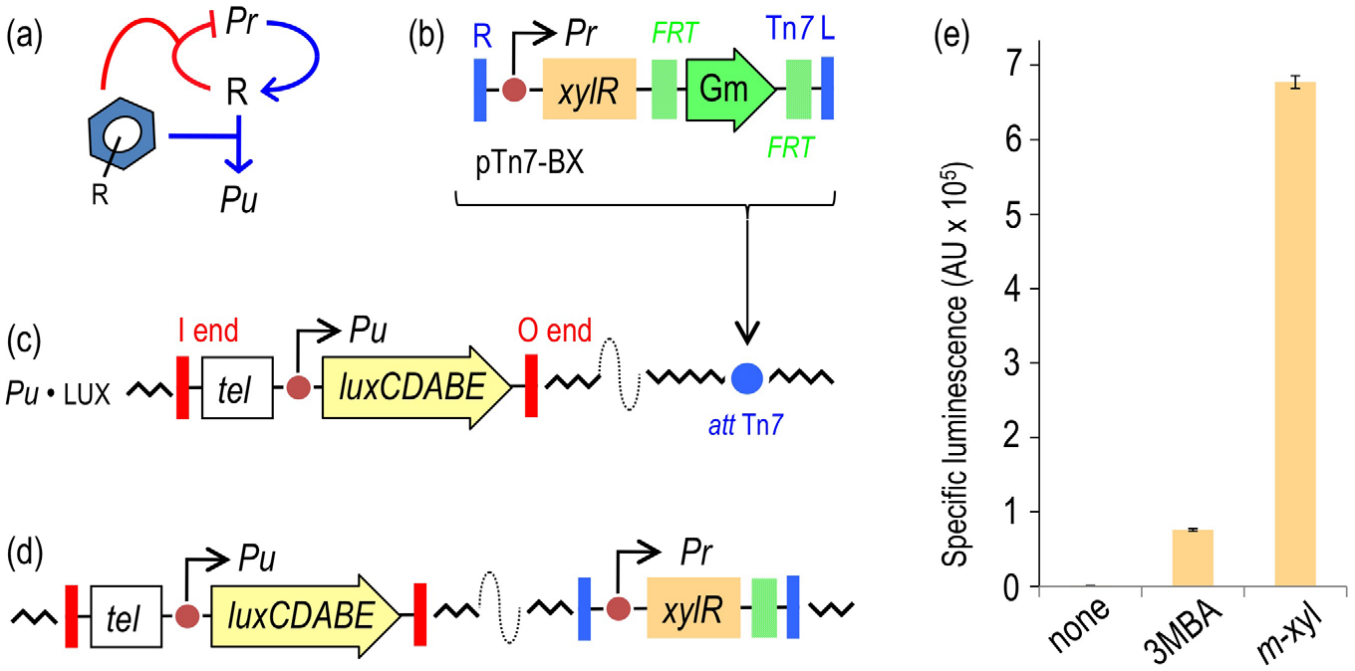
Performance of the XylR/*Pu* regulatory node in response to optimal (*m*-xylene) and suboptimal (3-methylbenzyl alcohol, 3MBA) inducers. (a) Relational map of the components of the node. The device includes the *Pr* promoter for *xylR* (R) transcription, the *Pu* promoter activated by XylR and the aromatic effector. Note that the inducer both triggers the action of XylR on *Pu* and increases transcriptional auto-repression of the TF. (b) The mini-Tn*7* element carrying *xylR* under the control of its native promoter *Pr* borne by the delivery plasmid pTn*7*-BX is inserted into the *att*Tn*7* site of a destination strain by means of selection for Gm^R^. (c) *P. putida* Pu·LUX carries a chromosomal insertion of a Tel^R^ mini-Tn*5* transposon with a transcriptional fusion *Pu*-*luxCDABE*. (d) Following insertion of the mini-Tn7 element, the Gm^R^ marker is eliminated upon transient expression of the yeast flippase, thereby generating the reference reporter strain *P. putida* BX. (**e**) Specific bioluminescence produced by *P. putida* BX in response to saturating vapors of *m*-xylene or 1 mM 3MBA (see Materials and Methods for details).

As shown in Figure 1, the event that triggers the regulatory and metabolic program encoded in the TOL plasmid is the binding of the pathway substrate to the XylR protein [7] and the ensuing activation of the *Pu* promoter for expression of the *upper* operon [6]. This results from the interplay of four components: [i] the aromatic effector, [ii] the *Pr* promoter that transcribes *xylR*, [iii] the XylR protein itself and [iv] the *Pu* promoter that is targeted by XylR. Although many other host factors influence the activity of the system *in vivo* [14], [21], [22], the set comprising the inducer/*Pr*/XylR/*Pu* forms the master regulatory device that determines signal specificity, i.e., the responsiveness of *Pu* to different aromatic effectors [11], [23]. The relational map of this node is depicted in Figure 2a. Exposure to aromatic effectors gives rise to a form of the XylR protein that both activates *Pu* and represses *Pr*, i.e., downregulates its own transcription. To have a reliable test system for comparing the inputs and outputs associated with this node, we engineered these components in a strain of *P. putida* bearing a transcriptional *Pu-luxCDABE* fusion inserted into its chromosome via a mini-transposon vector (*P. putida Pu*·LUX, Figure 2c). To ensure a faithful comparison of the input (i.e., inducer) and output (light emission) transfer function for each of the configurations tested, we assembled *xylR* expression in a specialized plasmid called pTn*7* Gm FRT [24]. This vector targets any DNA segment inserted therein to a natural *att*Tn*7* site present in the genome of *P. putida* KT2440 [25] in a specific orientation. Furthermore, once inserted, the Gm resistance marker can be excised through site-specific recombination between two flanking FTR sequences, thereby leaving the cells free of antibiotic resistances. To set a benchmark for the subsequent studies, we first produced a strain with the *Pr*/XylR/Pu regulatory parts connected in the same configuration as the natural TOL plasmid (Figure 2a). To this end, a DNA segment encoding the *xylR* gene placed under its native promoter was assembled in the aforementioned Tn*7* vector to yield pTn7-BX (Figure 2b). The insert was then delivered to the *P. putida* Pu·LUX chromosome and the Gm marker was deleted as shown in Figure 2c and 2d, thereby generating *P. putida* BX, which was thereafter the reference reporter strain. Note that all subsequent strains handled below carry the *Pu-luxCDABE* already described.

**Figure 3:**
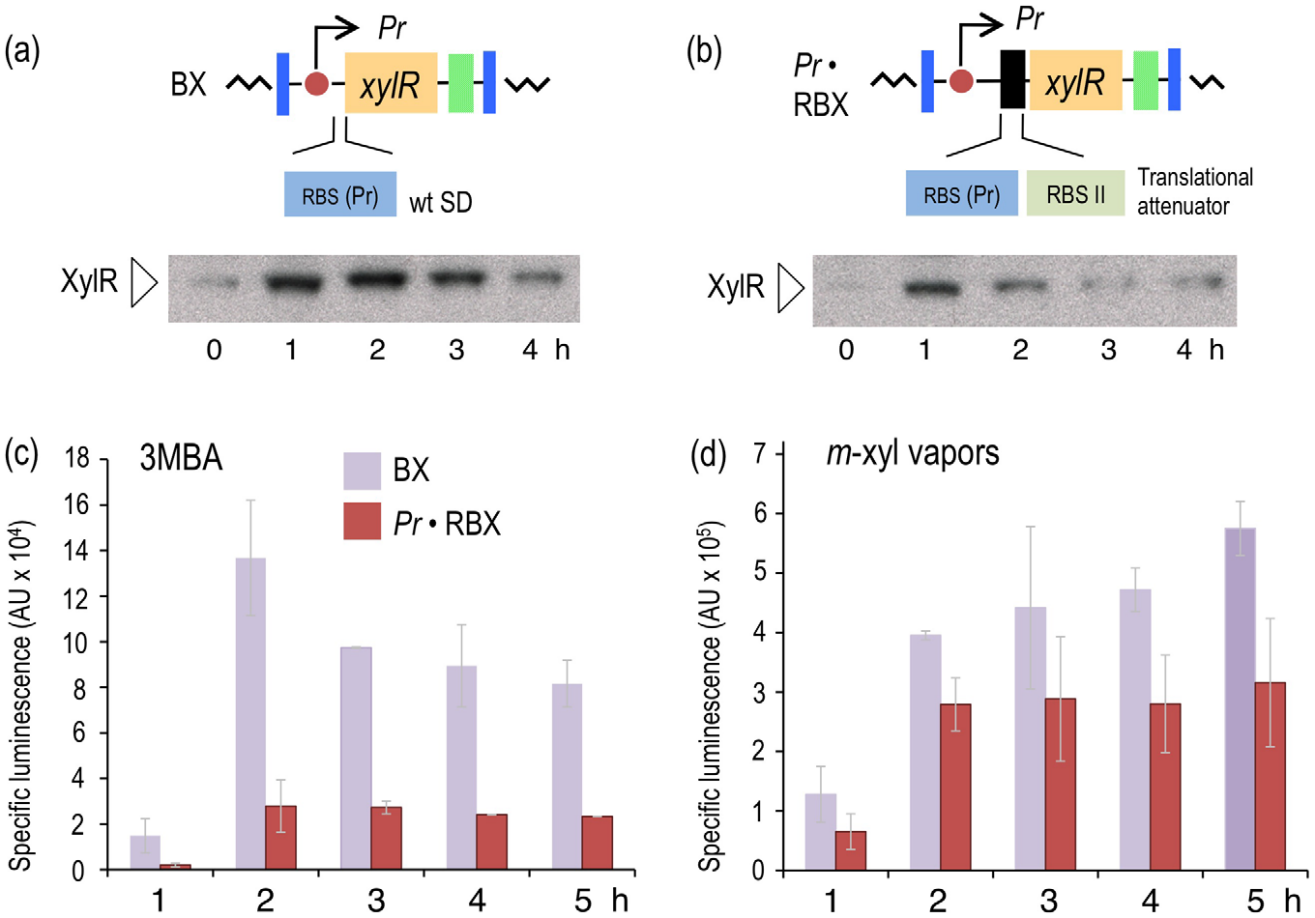
Effect of translational attenuation on performance of the XylR/*Pu* regulatory node. (a) Intracellular XylR levels under reference conditions. The image on top (not to scale) indicates the regulatory parts of the hybrid mini-Tn*7* element borne by *P. putida* BX, which transcribes *xylR* under the control of its natural *Pr* promoter and its native RBS. The Western blot (below) developed with an anti-XylR antibody indicates the relative concentration of cellular XylR over time. (b) Intracellular levels of translationally attenuated XylR in the *P. putida* Pr·RBX strain. The top image depicts how the 5′-UTR of xylR has been modified to reduce TF translation [28], as documented in the Western blot (below). (c) Specific bioluminescence produced by *P. putida* BX and *P. putida* Pr·RBX in response to 1.0 mM 3MBA over time. (d) As in (c), but induced with saturating vapors of *m*-xylene.

To quantify the response of the regulatory node of Figure 2a implemented in *P. putida* BX, the strain was grown in liquid medium and exposed to either optimal inducer vapor (*m*-xylene) or to 1.0 mM of a suboptimal effector (3MBA) and the resulting bioluminescence was recorded 5–6 h post-addition. This timing does not significantly affect luminescence (e.g. see Figure 3 below). While the background reading of the *Pu* output was in the range of 10^3^ luminescence units/OD_600_, the *m-*xylene-induced levels were close to 10^6^ (Figure 2e). These results confirmed the inducibility and strength of the *Pu* promoter and set a minimum and maximum window of activity for the rest of the work. The addition of 3MBA in the assay produced a luminescence readout that was only approximately 15% of that obtained with the optimal XylR effector but still very high relative to the background, non-induced levels. Such a difference between one inducer and the other is not understood mechanistically, as the apparent binding affinities of both good and bad XylR inducers are similar [26]. In either case, it is plausible that the output of the sensing device as a result of induction by either aromatic compound is limited by the intracellular concentrations of XylR [13], which curbs the robustness and sensitivity of the system [3], [4]. With this background, we wondered whether we could exacerbate the difference between optimal and non-optimal inducers (and thus increase signal specificity) by artificially increasing some of the parameters of the existing node (Figure 2a), by rewiring its connections or by both methods. Various approaches to this goal are explained below.

### Translational attenuation of XylR decreases the response of *Pu* to 3MBA

One theoretical way to modify the sensitivity of a signaling route involves changing the levels of the proteins involved in the process [27]. With this in mind, we entertained the possibility that lowering XylR concentrations could suppress the response of the regulatory device (Figure 2a) to 3MBA while preserving the induction of the same system by *m-*xylene. To test this, we constructed a variant of the node that kept the same relational organization but caused a drop in the levels of XylR by attenuating the protein's translation with tandem, repeated non-overlapping RBSs (Figure 3b) [28]. This modification was expected to lower translation of the downstream ORF without any effect on mRNA stability [29]. To implement this change, the Tn*7* plasmid pTn7-Pr·RBX was built as explained in the Materials and Methods section and delivered into *att*Tn*7* of *P. putida* Pu·LUX, as explained previously. The resulting strain, *P. putida* Pr·RBX, was identical to the reference strain *P. putida* BX except for a translational attenuator at the 5′-upstream untranslated region (5′-UTR) of the *xylR* gene (Figure 3b).

We next verified that the changes in the (UTR) of *xylR* lowered the net expression levels of the regulator without affecting its production kinetics. Thus, in parallel, we grew strains *P. putida* BX and *P. putida* Pr·RBX in LB medium and exposed them to 1.0 mM of the suboptimal effector 3MBA. Then, we performed Western blot analyses of the cell extracts with an anti-XylR antibody at various times after induction (Figure 3a and 3b). The pattern of induction in the strain with the wild-type 5′-UTR (*P. putida* BX, Figure 3a) was such that expression of XylR reached a maximum during the period 1–2 hours after exposure to the inducer, followed by a decrease at longer times, which was expected from the negative feedback loop that governs *xylR* expression (Figure 2a). The evolution of XylR in the counterpart strain bearing the modified 5′-UTR (*P. putida* Pr·RBX, Figure 3b) developed similarly, but the net concentration of XylR per cell was clearly lower. To examine the consequences of the different levels of the regulator in the response of the *Pu-luxCDABE* reporter to the suboptimal inducer, 3MBA was added to cultures of *P. putida* BX and *P. putida* Pr·RBX as before and their luminescence measured over time (Figure 3c). The results indicate that the overall output of the strain that expresses lower amount of XylR (*P. putida* Pr·RBX) was 4–7-fold lower than the strain carrying *xylR* controlled by its natural upstream region (*P. putida* BX). As a control, we also measured the response of the two strains with the different *xylR* 5′-UTRs to the optimal effector, *m*-xylene. As shown in Figure 3d, *Pu* output in the *xylR* 5′-UTR-modified strain *P. putida* Pr·RBX displayed a similar trend (although at somewhat lower levels) than the reference counterpart *P. putida* BX. However, we observed that the response of the cells to each inducer was more divergent in strain *P. putida* Pr·RBX than in strain *P. putida* BX. These results suggested that decreasing concentrations of XylR had the effect of widening the relative gap between the induction caused by 3MBA and *m-*xylene. Yet, the change in the *xylR* 5′-UTR was insufficient to entirely suppress the response of *Pu* to the suboptimal inducer. Moreover, lower XylR levels also caused a low-capacity regime with the optimal effector. Therefore, the next question was how to keep and even enhance *Pu* readout in response to *m-*xylene while removing the effect of 3MBA.

### Engineering a positive feedback loop (PFL) for augmenting the output of the *Pu*/XylR regulatory node

As shown in Figure 3d, the data indicate that decreasing intracellular XylR by changing the *xylR* 5′-UTR sequence caused a reduction of P*u* response to *m*-xylene by approximately 50%. It is thus plausible that the intracellular levels of the regulator determine the capacity (i.e., the maximum output) of the promoter. As the intracellular level of XylR under its native transcriptional control [13] is small and tends to decrease upon induction with aromatic effectors ([10] and Figure 3a and 3b), we wondered how making *xylR* transcription subject to a PFL (instead of the extant negative auto-regulation, Figure 2a) could affect the sensitivity and the capacity of the regulatory node to *m-*xylene and 3MBA. Positive auto-regulatory loops are prone to off/on expression patterns [30], [31] in a very TF concentration-sensitive fashion [32], [33]. We reasoned it would be possible to find a window of *xylR* expression that could trigger the on state with the optimal effector and leave the loop with 3MBA in the off state.

**Figure 4:**
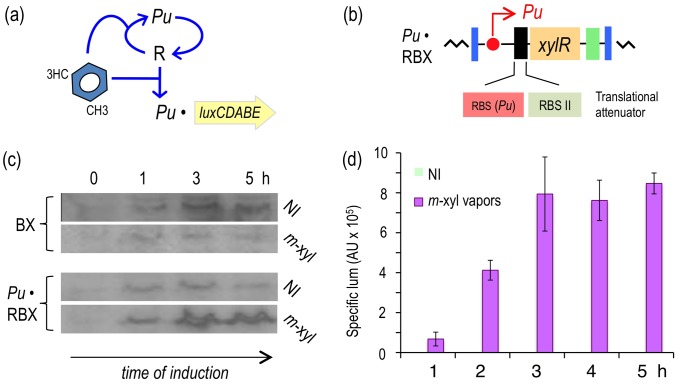
Subjecting expression of XylR to a positive feedback loop (PFL) based on *Pu*. (a) Relational map of the PFL. Unlike the connectivity of the components of the native node, the engineered regulatory loop has *xylR* transcribed through the XylR-responsive *Pu* promoter, which creates a device that becomes auto-induced in the presence of the aromatic effector. (b) Insert encoding the PFL in *P. putida* Pu·RBX. This strain carries a mini-Tn*7* with the *xylR* gene controlled by the *Pu* promoter and a translational attenuator formed by two tandem repeated ribosome binding sites: RBS(*Pu*) and the optimized RBSII [28]. (c) Western blot of *P. putida* BX and *P. putida* Pu·RBX extracts following exposure (or not) to *m*-xylene and probed with an anti-XylR antibody. (d) Specific bioluminescence produced by cultures of *P. putida* Pu·RBX exposed or not to *m*-xylene vapors, as indicated.

The first attempt in this direction involved the replacement of the native *xylR* promoter (*Pr*) by *Pu*, the promoter that is triggered by effector-bound XylR (Figure 4a). To this end, the same *Pu* sequence employed to construct the *Pu-luxCDABE* reporter was amplified with PCR primers and placed in front of a promoterless *xylR* gene preceded by the modified 5′-UTR [28] discussed above. The resulting expression device was then inserted at the *att*Tn7 site of the reporter *P. putida* chromosome (Figure 4b) [25], as described in the Materials and Methods to generate *P. putida* Pu·RBX. Note that this strain is entirely isogenic to *P. putida* Pr·RBX except that the *xylR* gene is expressed through *Pu* and not through *Pr*. This configuration changes the connectivity of the *Pu*/XylR node from a negative auto-inhibition device (Figure 2a) to a PFL (Figure 4a). To verify that such a modification in fact transforms the expression pattern of XylR *in vivo*, we used a Western blot to assay the accumulation of the protein in the reference strain (*P. putida* BX) and in *P. putida* Pu·RBX in the presence and absence of *m-*xylene. As shown in Figure 4c, the non-induced *P. putida* BX expressed XylR at low levels with a tendency to accumulate at later growth stages [34]. As expected, exposure to *m-*xylene under the same conditions resulted in lower XylR levels that appeared to decrease over time. The situation with the strain engineered with a forward loop (*P. putida* Pu·RBX) was similar under non-induced conditions but entirely different when cells were exposed to *m-*xylene. As shown in Figure 4c (bottom), the intracellular concentration of XylR quickly increased after one hour of induction, reaching very high levels at later growth stages. This experiment demonstrated not only that the positive loop engineered for expression of *xylR* worked as predicted but also that the effect of *m-*xylene on *Pu* was enough to switch the state of the loop to from low to high activity (see 0 h *vs.* 5 h of Figure 4c).

To examine whether expressing *xylR* through such an artificial regulatory device was translated into a high-capacity XylR/*Pu* node regime, we quantified the luminescence emitted by cultures of *P. putida* Pu·RBX induced with *m*-xylene vapors. As shown in Figure 4d, despite sustaining an attenuated translation of *xylR* because of the modified 5′-UTR introduced into this strain, the readout of *Pu* activity in *P. putida* Pu·RBX was as high as in the strain with the wild-type node (*P. putida* BX, Figure 2e) and more than twofold greater than the strain with the modified 5′-UTR but with wild-type regulatory connectivity (*P. putida* Pr·RBX, Figure 3d).

### Response of *P. putida* Pu·RBX to 3MBA

**Figure 5:**
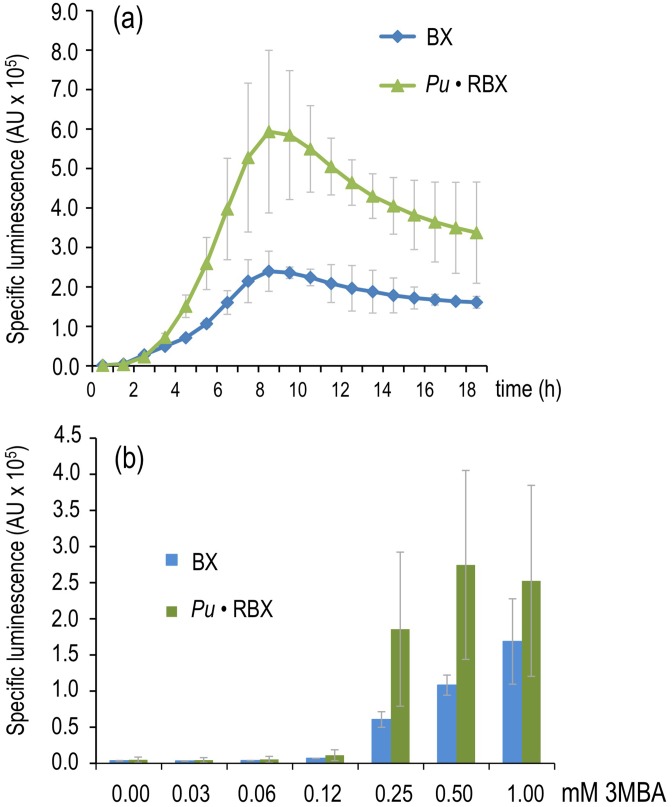
Effector sensitivity of strains expressing *xylR* through a negative or positive feedback loop. (a) Specific bioluminescence emitted by *P. putida* BX and *P. putida* Pu·RBX following addition of 1.0 mM 3MBA. (b) As in (a), but with *P. putida* BX and *P. putida* Pu·RBX cultures 6 hours after adding different concentrations of 3MBA as indicated.

The result of the creation of the construct discussed above was a circuit (Figure 4a) that responded to the *bona fide* XylR inducer (*m*-xylene) with a transcriptional strength comparable to the wild-type (Figure 2a) because the lower level of XylR caused by translational attenuation had been compensated for by a PFL. However, what is the effect of such a change on effector specificity? To examine this question, we monitored the luminescent response of a culture of *P. putida* Pu·RBX to 3MBA over time (Figure 5a) as well as the sensitivity of the same cells to increasing concentrations of this suboptimal inducer (Figure 5b). As a control, we employed the strain bearing the wild-type architecture of the regulatory node (*P. putida* BX). The results of our experiments (Figure 5) indicate that the response in the PFL-engineered strain to 3MBA was twofold greater than the response of the wild-type construct. This magnification is expected in such PFL regulatory motifs, which are prone to amplify the response to the trigger signal once it reaches a given threshold [30], [32]. This scenario was confirmed by the results shown in Figure 5b in which the responses of *P. putida* Pu·RBX and *P. putida* BX to 3MBA were followed along with moderate incremental increases of inducer concentrations. While *Pu* activity derived from the wild-type regulatory motif was only gradually increasing at 3MBA concentrations beyond 0.12 mM, the equivalent PFL strain displayed an abrupt change of *Pu* activity in cultures with 0.12 mM inducer (*Pu* very low) *vs.* those with 0.25 mM (*Pu* high to very high) and above. This phenomenon likely reflects the switch-on typically caused by the passing of a threshold in auto-inducing regulatory loops [32], [35].

While the results noted in Figure 5 did not by themselves elucidate the fundamental mechanism underlying our primary question of interest (discrimination of two chemically related inducers of *Pu*, see above), they demonstrated that the response of the XylR/*Pu* node to inducer could be made more digital in a fashion dependent on the concentration of the TF involved. These results suggested that one could keep the node in an entirely off state when XylR levels are below a certain threshold, while triggering a high activity regime once the threshold has been surpassed. On this basis, we recreated the same PFL but pursued a higher limit for XylR auto-induction in a way such that optimal and suboptimal effectors could trigger or not, respectively, a high-activity of the downstream *Pu* promoter.

### A *Ps* promoter-based PFL enhances activity and specificity of the XylR/*Pu* regulatory node

**Figure 6:**
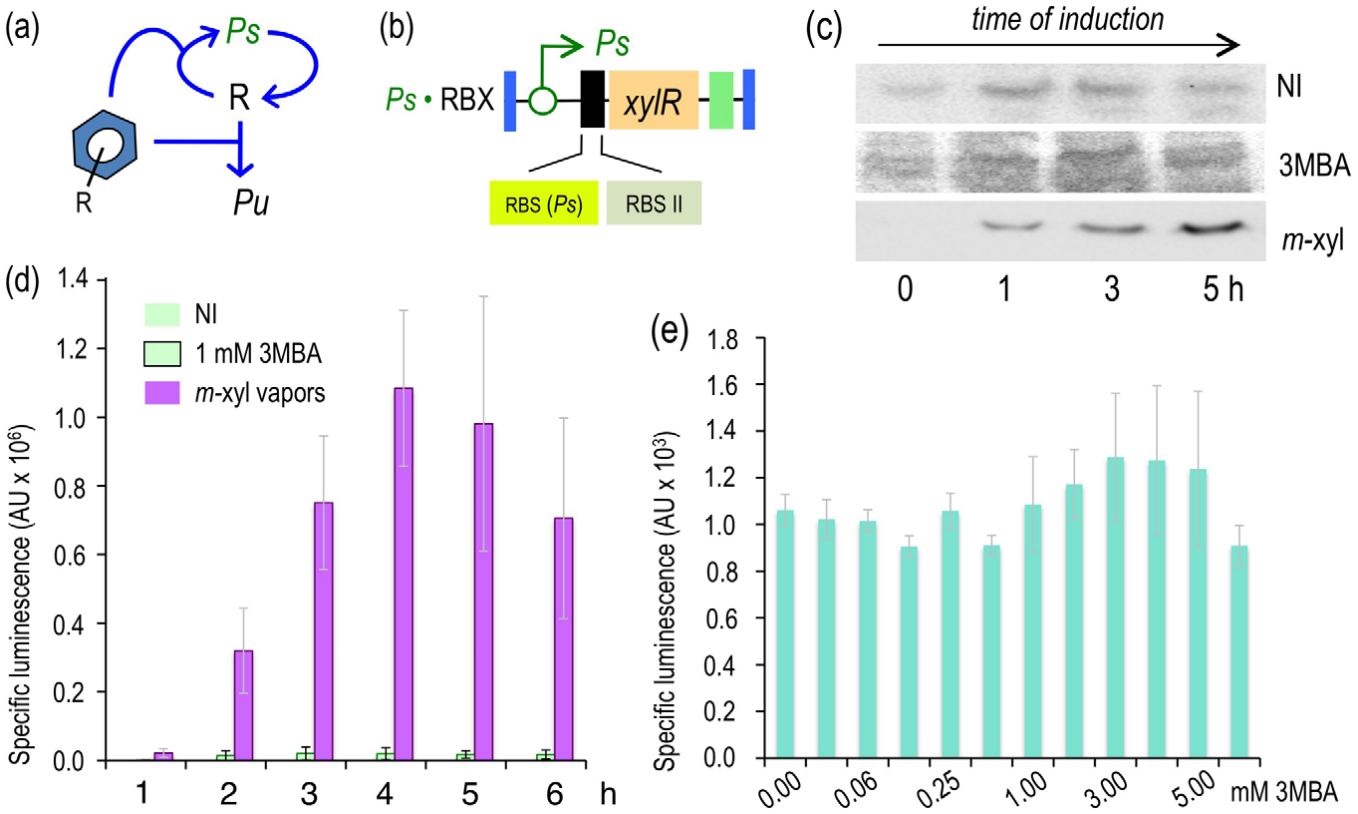
Induction of the XylR/*Pu* node in strains engineered with a *Ps*-PFL for *xylR* expression. (a) Relational map of the *Ps*-PFL device. The regulatory loop has *xylR* transcribed through the XylR-responsive but relatively weaker *Ps* promoter. (b) Insert encoding the *Ps*-PFL in *P. putida* Ps·RBX. This mini-Tn*7* has the *xylR* gene controlled by the *Ps* promoter and a translational attenuator formed by two tandem repeated RBSs, as indicated. (c) Western blot of *P. putida* BX and *P. putida* Ps·RBX cells following exposure (or not) to *m*-xylene or 3MBA, as indicated, and probed with an anti-XylR antibody. (d) Specific bioluminescence produced by cultures of *P. putida* Ps·RBX exposed to *m-*xylene vapors or 1.0 mM 3MBA, as indicated. (e) Sensitivity of *P. putida* Ps·RBX to 3MBA. The graph displays the specific bioluminescence emitted by cultures of the strain 6 hours after addition of different concentrations of 3MBA as indicated.

The results above indicate that making XylR expression subject to a PFL increases the amplitude of the XylR/*Pu* response to both optimal (*m*-xylene) and suboptimal effectors (3MBA), which means that both effectors cause XylR (controlled by the attenuated *Pu-*based PFL) to reach the TF threshold imposed by this auto-inducing architecture (Figure 4a) [30]. The subsequent question was whether suppression of any response to 3MBA could be brought about by moving the window of effector-induced *xylR* transcription in the PLF to a range that could still trigger full response to *m-*xylene but remain impervious to the suboptimal inducer. To check this, we simply replaced the *Pu* promoter of the *P. putida* Pu·RBX (Figure 4a) with a weaker but still XylR-responding promoter, *Ps* of the TOL plasmid [36]. As XylR activates *Ps* in response to aromatic effectors at a lower level than *Pu* [37], we hypothesized that a *Ps*-based PFL would make the switch-on threshold more difficult to reach for a suboptimal inducer. We constructed a *P. putida* strain (Figure 6a) placing *xylR* and the RBS II [28] downstream of the regulatory region of the *xylS* gene including its own RBS(*Ps*), using the same methods employed for construction of other strains (see Materials and Methods). This new strain, which was engineered with a *Ps*-based PFL (Figure 6a), was named *P. putida* Ps·RBX (Figure 6b). To examine the response of the new regulatory loop of this strain to either effector, *P. putida* Ps·RBX was grown in the absence or presence of each aromatic inducer and the intracellular levels of XylR recorded over time along with light emission. Figure 6c reveals that 3MBA failed to trigger the auto-activation loop for XylR expression, suggesting that the levels of the TF were insufficient to switch on the PFL. Consistent with those results, 3MBA also failed to cause any significant activation of the downstream *Pu-luxCDABE* reporter (Figure 6d). This situation did not change when more inducer was added to the culture (Figure 6e), thereby confirming that the silencing of the PFL could be traced to nothing else but XylR. In contrast, when the same *P. putida* Ps·RBX cells were induced with *m-*xylene, the cells exhibited a noticeable accumulation of the XylR protein over time (Figure 6c) as well as a strong emission of light (Figure 6d). In fact, the output of the *Pu-luxCDABE* reporter was twofold higher than that of the wild-type regulatory node of *P. putida* BX (Figure 2e). These results indicated that, unlike the native effector-responding device of the TOL plasmid, the regulatory architecture implemented in *P. putida* Ps·RBX could discriminate between optimal and suboptimal inducers in a fashion that was not dependent on their concentration but on their chemical structure alone. Unfortunately, the very low levels of expression of XylR under this PFL made detection of intracellular XylR concentrations difficult in cells exposed to 3MBA (Figure 6c). The mechanistic basis of effector discrimination could therefore be inferred but not really proven. To overcome this uncertainty, we resorted to a further perturbation of the system as explained below.

### Increasing the basal expression of the *Ps*-based PFL restores innate promiscuity of the XylR/*Pu* node

**Figure 7:**
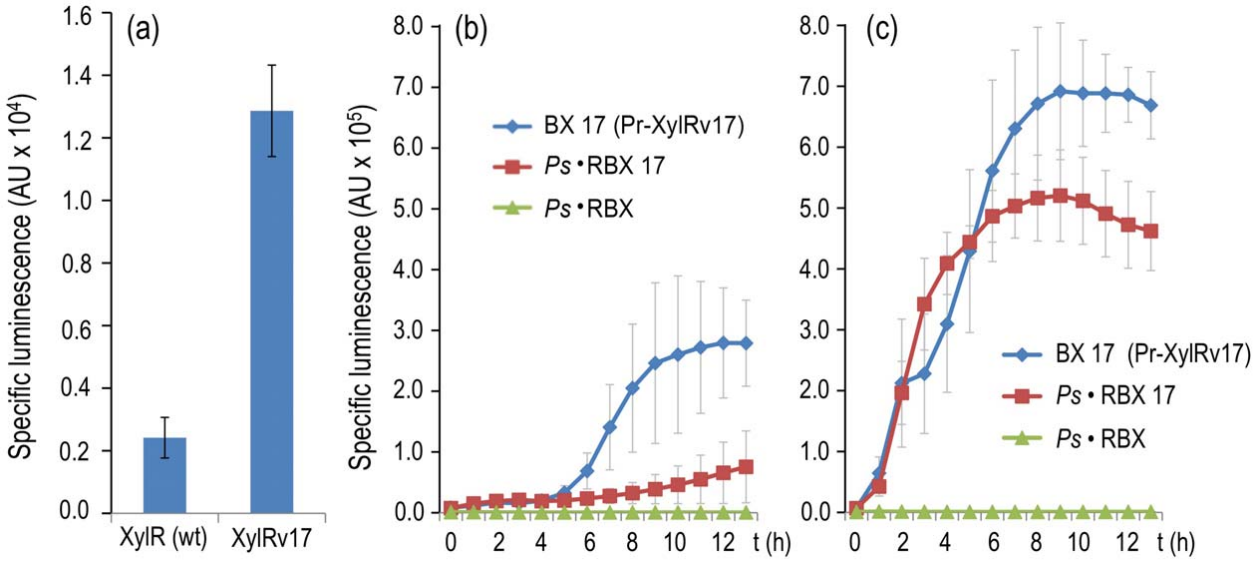
Breaking effector discrimination with a semi-constitutive variant of XylR. (a) Specific bioluminescence produced by cultures of *P. putida* BX (encoding wild-type XylR) and *P. putida* BX17 (encoding XylRv17) after 6 h of incubation in the absence of effectors. (b) Specific bioluminescence produced by cultures of *P. putida* BX17, *P. putida* Ps·RBX and *P. putida* Ps·RBX17 cultures over time without inducers. (c) Same, following addition of 1.0 mM of 3MBA. Note that the insensitivity of *P. putida* Ps·RBX to the suboptimal inducer is lost in the equivalent construct expressing XylRv17, which recovers a level of *Pu* output comparable to that of the wild-type.

The increase in the signal specificity of the XylR/*Pu* node reported above could be attributed to a change in the threshold necessary to trigger the response produced by the new regulatory loop of *xylR*. Should this be the case, any resetting of such a threshold back to its former sensitivity range is predicted to restore the response to the suboptimal effector, 3MBA. How can this be accomplished without varying the architecture of the node yet again? To solve this conundrum, we decided to replace the wild-type *xylR* sequence of the *Ps*-based PFL device with the variant *xylRv17*. This mutant encodes a XylR derivative that is responsive to all aromatic effectors of the wild-type protein, but it is also able to trigger low-level activity of target promoters in the absence of any inducer [17], [26]. The expected result of having *xylRv17* expressed under the control of a *Ps*-based PFL is therefore to downshift the threshold of active TF that is necessary for switching on the auto-inducing device. To test these predictions, we first constructed a control strain *P. putida* BX17, which was identical to *P. putida* BX except that the encoded TF sequence is *xylRv17* [26] instead of wild-type *xylR*. As shown in Figure 7a, *P. putida* BX17 displayed a basal *Pu* activity level in the absence of effectors ≥7-fold higher than that of the strain bearing the wild-type *xylR* gene. The test strain, in contrast, was the same as the *P. putida* Ps·RBX examined above, but *xylR* had been similarly replaced by *xylRv17*, thus giving rise to *P. putida* Ps·RBX17 (see the Materials and Methods for construction details). The only difference between the two is the minor semi-constitutive expression of *xylRv17* compared with the original TF. This disparity has, however, dramatic consequences in the sensitivity of the regulatory device as a whole to 3MBA. Figure 7b and 7c compares the emission of light of the *P. putida* BX17 (*xylRv17* under TOL plasmid *Pr* promoter), *P. putida* Ps·RBX (wild-type *xylR* expressed through the *Ps*-based PFL) and *P. putida* Ps·RBX17 (same but *xylRv17*) strains with and without 3MBA. The results indicate that *P. putida* Ps·RBX17 was nearly as responsive to this effector as the strain bearing the native configuration of the regulatory system. Consistent with the results reported above (Figure 6), no light emission above background levels could be detected from *P. putida* Ps·RBX under the same conditions. Taken together, these data strengthened the notion that up- or downshifting of the auto-activation threshold of the PFL by adjusting the concentration or activity of the TF resulted in a regulatory device whose specificity to given effectors could be drastically changed.

## Discussion

The regulatory networks that control gene expression in cells and organisms have evolved to accurately adjust their reactions to specific stimuli [38]. Traditionally, the well-characterized pieces of these regulatory networks haven been exploited to engineer cells with new responses. Although this approach was useful for constructing a plethora of strains presenting new phenotypes [15], [20], [39]–[41], it was not until the onset of systems and synthetic biology that we began to understand how the output of a specific circuit was conditioned by the shape of the network in which the parts are interconnected. Thus, bottom-up approaches shed light on the intrinsic properties of regulatory networks, allowing for the rational design of newly engineered genetic circuits [3], [42]. Prokaryotic regulatory systems have been used in the construction of bacterial strains with biotechnological applications, such as whole-cell biosensors to detect environmental pollutants [15], [41], [43], [44]. Such biosensors are generally based on the association of input/output components that usually include one bacterial transcriptional regulator that acts as a sensor module and a reporter gene coupled to its cognate promoter [39], [45]. The specificity of engineered regulatory networks primarily relies on the responsiveness of the transcriptional factor to the signal of interest [41]. Based on this understanding, the quest for new signal specificities has been generally based on *in vitro* modification of the sensor module [17], [18], [26], [46]. In this work, we have demonstrated that, by rational rewiring of the architecture of a specific regulatory network, it is possible to modify the input-output function to increase the amplitude or the specificity of the response without modifying the core sensor part of the circuit. To this end, we took advantage of the well-characterized TOL network of *P. putida* pWW0 [7], where XylR controls the expression of several genes by binding two target promoters, *Pu* and *Ps* [6]. As shown in Figure 2, XylR responds strongly to *m*-xylene and, to a lesser extent but still significantly, to 3MBA. As shown above, by modifying the connectivity of the components of the regulatory system, we could [i] increase the general amplitude of the output and [iii] generate a super-specific response to the optimal inducer by filtering the response to the less favorable XylR effector. To accomplish this, we first considered simply lowering the concentration of some of the components of the regulatory system, with the aim of increasing the activation threshold and thus increasing specificity [47]. This approach is not without precedent, as previous studies indicate that controlling the expression levels of MAP kinases in regulatory cascades, through gene expression or post-translational modifications, make it possible to change the activation profile of the system [27]. However, this did not suffice for discrimination between optimal and suboptimal effectors (Figure 3c and 3d). In the natural and translationally attenuated context, the levels of XylR are maintained within limits through a negative feedback loop mediated by the *Pr* promoter (Figure 1; [10], [34]). We demonstrated above that replacing this negative auto-regulation by a PFL leads to an amplification of the system output in a fashion typical of bistable switches [30], [31], [47]. Furthermore, the combination of a translational attenuator with PFLs endowed with different auto-induction parameters resulted in regulatory devices with activation thresholds far enough apart to discriminate between the two XylR effectors tested. Although other approaches have been used to increase signal sensitivity [19], [48], this is, to the best of our knowledge, the first instance that modifies the specificity of a sensor system by simply rewiring the connectivity of the parts involved. We argue this approach is extraordinarily promising for improving the performance of whole-cell biosensors [49], more so when combined with modification of the core TF [17] or the output modules [28], [50] in the design of optimized devices.

**Figure 8:**
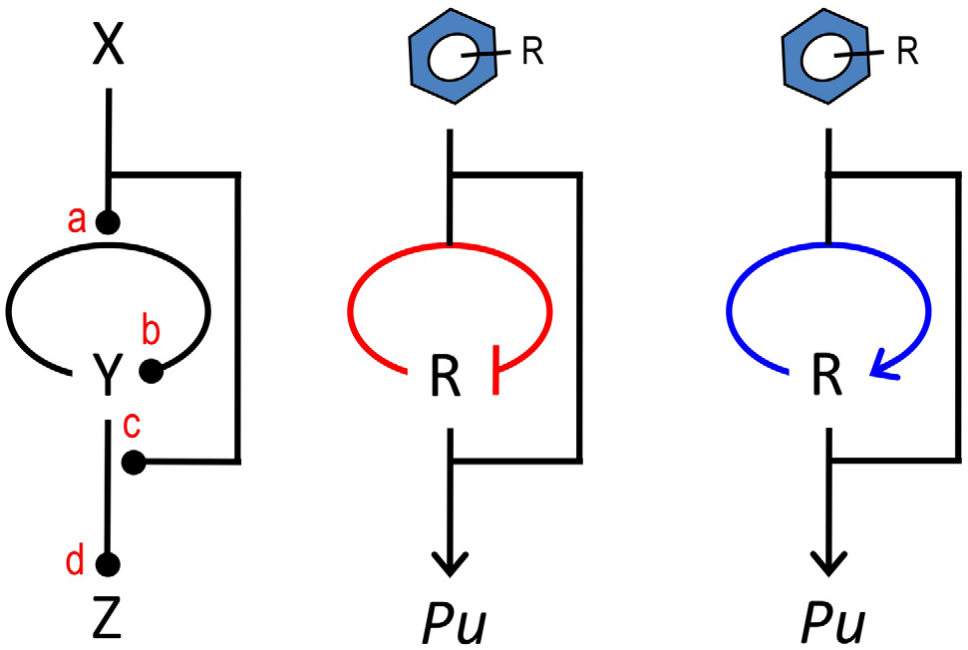
The master control loop (MCL). The sketch to the left shows a common arrangement of regulatory elements in devices that control expression of pathways for biodegradation and detoxification of environmental pollutants. The motif involves an upstream signal (the effector X) that influences expression of a cognate regulator Y that, in turn, binds the inducer X for acting on a target promoter Z. The motif has 4 transfer functions (a, b, c, d) that can be combined to produce a large number of regulatory possibilities. In this work, we documented that changing the sign of the auto-regulation loop that governs *xylR* expression from its native negative architecture (middle) to a positive interaction (right) causes the system to discriminate between an optimal and a suboptimal effector of the system.

Finally, we have not failed to observe that a large number of regulatory nodes for biodegradative and detoxification operons [51] follow a general architecture that we have designated the *master control loop* (MCL, Figure 8). This theme, which is also implicit in many metabolic and regulatory networks [52] consists of an upstream signal (i.e., the metabolic substrate or effector) that both influences expression of the cognate regulator as well as the interaction of the same TF with the downstream target promoter. The 3 components of the motif can interact at 4 sites of the related object and present up to 16 theoretically possible configurations. The native arrangement of the TOL regulatory network, as well as those that have been engineered for the sake of this work, are simply variants of such a general layout. The work above suggests that this motif is endowed with extraordinary plasticity for responding to the specifications of any given regulatory need in terms of capacity, inducibility and signal specificity. We propose such an MCL motif as a frame of reference for the further development of regulatory devices *á la carte*, as required in contemporary metabolic engineering and other fundamental and biotechnological applications.

## Materials and Methods

### Strains, culture conditions, and general procedures

*P. putida* KT2440 [53], its derivatives and the *E. coli* strains used in this study were grown in Luria-Bertani (LB) medium and handled with standard procedures. *E. coli* CC118λ*pir* was used as the host for propagating plasmids based on an R6K origin of replication [54]. When required, the media was amended with the specified concentrations of 3-methylbenzylalcohol (3MBA) or saturating vapors of *m*-xylene. Antibiotics were used at the following concentrations: piperacillin (Pip) 40 µg/ml, chloramphenicol (Cm) 30 µg/ml, gentamycin (Gm) 10 µg/ml, tetracycline (Tc) 10 µg/ml and potassium tellurite (Tel) at 80 µg/ml. PCR reactions were performed as follows: 50–100 ng of the template indicated in each case was mixed in a 100-µl reaction mixture with 50 pmol of each of the primers specified and 2.5 units of Pfu DNA polymerase (Stratagene). The samples were then subjected to 30 cycles of 1 min at 95°C, 1 min at 58°C and 3 min at 72°C. Clones were first verified by colony PCR [55] using 1.25 units of Taq DNA polymerase (Roche) and later confirmed with DNA sequencing. Other gene cloning techniques and standard molecular biology procedures were performed according to [55].

### Construction of hybrid Tn*7* delivery plasmids

DNA segments containing the *xylR* gene under the control of different promoter architectures were cloned in vector pTn7-Gm FRT [24] for their eventual insertion at the native *att*Tn*7* site of the *P. putida* chromosome [25]. Such insertions occur always at the same site and in the same orientation, thus generating entirely equivalent strains [24]. To construct the corresponding mini-Tn*7* delivery vectors, we first engineered a series of pUC18Not derivatives [54] carrying the DNA segments at stake as follows. A 2.8-kb *Kpn*I-*Sac*I fragment of pBBXylR [56] containing the construct *Pr → xylR* (i.e., the *xylR* gene expressed through its native promoter of the TOL plasmid) was inserted in a pUC18Not variant that lacked the *Eco*RI site, thereby generating pBXe. This plasmid was used as the frame for replacing the native promoter region of *xylR* with the 3 alternative 5′-upstream sequences employed in this work. In the first case, *P. putida* mt-2 genomic DNA was amplified with the primersPR 1F (5′-CGctcgagGTTAACATAATCGGAGACTGC-3′) and PRrbs 2R (5′-CCGgaattc*CAT*GCTTAATTTCTCCTCTTTTTGTTT**TCCT**CTTGTTTTTAT-3′). The resulting 545-bp product contained the native *Pr* promoter and the adjacent sequence down to the natural RBS (bold) but with an added RBSII (underlined in the sequence [28] between the original RBS and the cognate ATG codon in italics). Furthermore, the amplified segment was flanked by the *EcoR*I and *Xho*I restriction sites introduced in the primers (lower case in the sequences above). In the second case, primers PU 1F (5′-ACGCctcgagCCCGGGAAAGCGCGATGA-3′) and PUrbs 2R (5′-CGgaattc*CAT*GCTTAATTTCTCCTCT TTTGAAGGG**TCACC**ACTATTTTT-3′) amplified a 464-bp segment containing *Pu* all the way down to the RBS of the *xylU* gene (bold), which was then followed by the native RBSII (underlined) and ATG (italics) of the *xylR* gene, flanked by *EcoR*I and *Xho*I sites (lower case). Finally, the primers PS 1F (5′-CGctcgagTTGTTTTCCTCTTGTTTTTATCG-3′) and PSrbs 2R (5′-CGgaattc*CAT*GCTTAATTT CTCCTCTTTAGTTCACGGT**TCTC**TTATT**-**3′) resulted in a *EcoR*I-*Xho*I 256-bp fragment containing the second XylR-responsive promoter *Ps* of the TOL plasmid (Figure 1) down to the RBS of the *xylS* gene (bold) and followed by the RBSII (underlined) and ATG (italics) of the *xylR* gene as before. Each of these 3 *EcoR*I-*Xho*I restriction products were cloned into the corresponding sites of the pBXe plasmid, thereby replacing the original *xylR* upstream region with refactored counterparts and originating pPrRBX, pPuRBX and pPsRBX. These plasmids were separately digested with *Not*I, which excised DNA segments carrying *Pr → xylR, Pr-(RBS_Pr_ RBSII) → xylR, Pu-(RBS_Pu_ RBSII) → xylR and Ps-(RBS_Ps_ RBSII) → xylR*. These were cloned in the same orientation into the *Not*I site of pTn*7*-Gm FRT [24] generating pTn*7*-BX, pTn7-Pr·RBX, pTn7-Pu·RBX and pTn7-Ps·RBX. For the constructs bearing the semi-constitutive XylR variant named XylRv17 (which carries mutations F48I and L222R; [17]), a 713-bp *EcoR*I-*Avr*II fragment-spanning DNA sequence corresponding to the A domain of *xylRv17* was excised from plasmid pBB*xylRv17* [17] and recloned into the corresponding sites of pBXe or pPs·RBX, yielding pBX17 and pPs·RBX17, respectively. These plasmids were then digested with *Not*I and the fragments encoding *Pr → xylRv17* and *Ps-(RBS_Ps_ RBSII) → xylRv17* were cloned, as before, in vector pTn7-Gm FRT [24], thereby generating pTn*7*-BX17 and pTn7-Ps·RBX17.

### Strain construction

Standardization of the various regulatory devices for *xylR* expression and measurement of network output required the engineering of a reference *Pu-luxCDABE* reporter *P. putida* strain. To this end, we first digested plasmid p*att*PuLUX [24] with *EcoR*I/*Xma*I to delete an internal 86-bp fragment containing the *E. coli att*Tn*7* insertion site [57]. The ends of the digested plasmid were blunted with T4 DNA polymerase and relegated to generate the plasmid pPu·LUX. This construct was then digested with *Not*I, and the fragment containing a *Pu-luxCDABE* fusion ligated to pJMT6, a mini-Tn*5* delivery vector with a potassium tellurite (Tel) resistance cassette [58], producing pTn*5* Tel-Pu·LUX. This plasmid was conjugally transferred to *P. putida* KT2440 (see below) and Tel^R^ exconjugants tested for insertion of the hybrid mini-Tn*5* Tel element carrying the *Pu-luxCDABE* fusion. One of these clones was called *P. putida* Pu·LUX and retained for further use as receptor of the different variants of the mini-Tn7 transposons borne by the plasmids pTn*7*-BX, pTn*7*-Pr·RBX, pTn*7*-Pu·RBX, pTn*7*-Ps·RBX (see above). Insertion of the corresponding segments in the naturally occurring *att*Tn*7* site of *P. putida* Pu·LUX was confirmed through colony PCR using a primer that anneals within the sequence of the B domain of *xylR* (NDoCAvrII: 5′-GCGAATGGCCTAGG CCGTAATACTG-3′) and one at the *att*Tn*7* insertion site within the *glmS* gene (Ppu*glmS* 2R: 5′-GTGCGTGCCCGTGGTGG-3′). The ensuing collection of Gm^R^ strains were deleted of this antibiotic marker by transient expression of yeast flippase encoded by the plasmid pBBFLP, which brings about site-specific recombination of the FRT sequences that flank the resistance gene [56]. The same strategy was followed in the case of pTn7-BX17 and pTn7-Ps·RBX17, although the Gm^R^ marker was not removed in these cases. The final outcome of all these manipulations was the isogenic strain collection *P. putida* BX, *P. putida* Pr·RBX, *P. putida* Pu·RBX, *P. putida* Ps·RBX, *P. putida* BX17 and *P. putida* Ps·RBX17.

### Plasmid transfer and mini-transposon delivery into *P. putida*

pTn*5*Tel-Pu·LUX and mini-Tn*7* derivatives (pTn7-BX, pTn*7*-Pr·RBX, pTn*7*-Pu·RBX, pTn*7*-Ps·RBX, pTn*7*-BX17 and pTn*7*-PsRBX17) and pBBFLP were conjugally passed from the donor *E. coli* strain indicated in each case into the different *P. putida* recipients with a filter mating technique [54]. To this end, a mixture of donor, recipient and helper strain *E. coli* HB101 (pRK600) was deposited on 0.45-µm filters in a 1∶1∶3 ratio and incubated for 8 h at 30°C on the surface of LB-agar plates. Mini-Tn*7* derivatives were co-mobilized along with the transposase-encoding genes *tnsABCD* into the recipient strains by including *E. coli* CC118λ*pir* (pTNS1) in the mating mixture [59]. After incubation, the cells were resuspended in 10 mM MgSO_4_, and the appropriate dilutions plated on M9/succinate amended with suitable antibiotics for counter-selection of the donor and helper strains and growth of the *P. putida* clones that had acquired the desired insertions. *Bona fide* transposition was verified in every case by checking the sensitivity of individual exconjugants to the delivery vector marker, piperacillin.

### Bioluminescence assays

To measure bioluminescence production of *P. putida* cells carrying *luxCDABE* fusions, 2-ml cultures of the strains under study were first pre-grown in 10-ml test tubes overnight in LB medium at 30°C. The cultures were then diluted to an OD_600_ of 0.05 and grown up to an OD_600_ = 1.0 in 100-ml flasks. At this point, the cultures were exposed to *m-*xylene or 3MBA, as indicated for each case. When required, 200-µl aliquots of these cultures were placed in 96-well plates (NUNC), and light emission and OD_600_ were measured in a Victor II 1420 Multilabel Counter (Perkin Elmer). The specific bioluminescence values were calculated by dividing the obtained values of total light emission (in arbitrary units) by the optical density of the culture (OD_600_). The specific bioluminescence values shown represent the average of at least three biological replicates.

### Protein techniques

Protein analyses were performed according to published protocols [55]. For detection of the XylR (wild-type and variants), 5 µg of whole protein extract of *P. putida* cells was denatured in a sample buffer containing 2% SDS and 5% ß-mercaptoethanol and run on 10% polyacrylamide gels. These were subsequently blotted onto a polyvinylidene difluoride (PVDF) membrane (Immobilon-P, Millipore) using a semi-dry electrophoresis transfer apparatus (BioRad). After protein transfer, the membranes were blocked for 2 h at room temperature with MBT buffer (0.1% Tween and 5% skim milk in phosphate-buffered saline, PBS). For detection of XylR, the membranes were incubated with MBT buffer containing a dilution 1/2000 of anti-XylR Phab [13]. The membranes were subjected to 5-min washing steps in 40 ml of MBT buffer alone or MBT with 0.1% sodium deoxycholate in the case of the membranes hybridized with Phabs. To detect the anti-XylR Phab bound to the XylR bands, an anti-M13 peroxidase conjugate was utilized (1/5000 dilution in MBT). The membranes were incubated for 1 h at room temperature with a secondary antibody and washed 5 times in MBT buffer for 5 min each, as before. XylR was developed by reaction of the treated membrane with a chemiluminescent substrate (ECL, Amersham Pharmacia Biotech) and recorded on x-ray film.
